# Impact of computerized physician order entry on medication prescription errors in the intensive care unit: a controlled cross-sectional trial

**DOI:** 10.1186/cc3983

**Published:** 2006-01-26

**Authors:** Kirsten Colpaert, Barbara Claus, Annemie Somers, Koenraad Vandewoude, Hugo Robays, Johan Decruyenaere

**Affiliations:** 1 Medical Doctor, Staff Member, Intensive Care Department, Ghent University Hospital, Belgium; 2 Hospital Pharmacist, Staff Member, Pharmacy Department, Ghent University Hospital, Belgium; 3 Hospital Pharmacist, Staff Member, Pharmacy Department, Ghent University Hospital, Belgium; 4 Medical Doctor, Staff Member, Intensive Care Department, Ghent University Hospital, Belgium; 5 Professor in Pharmacy, Head of Pharmacy Department, Ghent University Hospital, Belgium; 6 Professor in Intensive Care, Head of Intensive Care Department, Ghent University Hospital, Belgium

## Abstract

**Introduction:**

Medication errors in the intensive care unit (ICU) are frequent and lead to attributable patient morbidity and mortality, increased length of ICU stay and substantial extra costs. We investigated if the introduction of a computerized ICU system (Centricity Critical Care Clinisoft, GE Healthcare) reduced the incidence and severity of medication prescription errors (MPEs).

**Methods:**

A prospective trial was conducted in a paper-based unit (PB-U) versus a computerized unit (C-U) in a 22-bed ICU of a tertiary university hospital. Every medication order and medication prescription error was validated by a clinical pharmacist. The registration of different classes of MPE was done according to the National Coordinating Council for Medication Error Reporting and Prevention guidelines. An independent panel evaluated the severity of MPEs. We identified three groups: minor MPEs (no potential to cause harm); intercepted MPEs (potential to cause harm but intercepted on time); and serious MPEs (non-intercepted potential adverse drug events (ADE) or ADEs, being MPEs with potential to cause, or actually causing, patient harm).

**Results:**

The C-U and the PB-U each contained 80 patient-days, and a total of 2,510 medication prescriptions were evaluated. The clinical pharmacist identified 375 MPEs. The incidence of MPEs was significantly lower in the C-U compared with the PB-U (44/1286 (3.4%) versus 331/1224 (27.0%); *P *< 0.001). There were significantly less minor MPEs in the C-U than in the PB-U (9 versus 225; *P *< 0.001). Intercepted MPEs were also lower in the C-U (12 versus 46; *P *< 0.001), as well as the non-intercepted potential ADEs (21 versus 48; *P *< 0.001). There was also a reduction of ADEs (2 in the C-U versus 12 in the PB-U; *P *< 0.01). No fatal errors occurred. The most frequent drug classes involved were cardiovascular medication and antibiotics in both groups. Patients with renal failure experienced less dosing errors in the C-U versus the PB-U (12 versus 35 serious MPEs; *P *< 0.001).

**Conclusion:**

The ICU computerization, including the medication order entry, resulted in a significant decrease in the occurrence and severity of medication errors in the ICU.

## Introduction

In 1999, the Institute Of Medicine reported that 44,000 to 98,000 people annually die in US hospitals as a result of medical errors [1]. Medication errors occurring either in or out of the hospital are estimated to account for at least 7,000 deaths each year [1]. Medication errors can occur in all stages of the medication process, from prescribing to dispensing and administration of the drug. Although most of these errors are harmless, or intercepted on time, some do result in an adverse drug event (ADE) [2-6]. According to Bates and colleagues [3,7], 1/100 in-hospital medication errors result in an ADE, and 7/100 have the potential to do so. Overall, 28% to 56% of all ADEs are judged preventable, and most of these errors occur in the ordering stage of the medication process [3,6,8,9]. It has been shown that the attributable cost ranges from $10 for a medication error without harm, to more than $5,000 for a serious ADE [10]. In intensive care unit (ICU) settings, the rate of preventable and potential ADEs is even higher, being almost twice as high as in non-ICUs [11]. This can be attributed to the high number of drugs that ICU patients receive, the preference for intravenous administration and the incidence of organ failure, all of which increase the potential for errors [11,12].

Studies published by the ADE Prevention Study Group indicate that prevention strategies targeting systems rather than individuals are more effective in reducing errors [13]. Computerized physician order entry (CPOE) has been recommended by the Leapfrog group as a major step to improve patient safety in the USA [10]. CPOE could eliminate many of the problems associated with manual drug order writing [1] by decreasing the occurrence of illegible orders, inappropriate doses and incomplete orders [14], which results in a substantial reduction in medication errors of 55% to 80% [7,15-17]. On the other hand, less sophisticated or older CPOE systems may have the potential to introduce new problems [18-22]. Until now, CPOE has never been shown to decrease patient morbidity or mortality [23], but seems to be especially helpful in preventing minor errors [17,22]. An intensive care information system (ICIS) is a computerized system specifically designed for the ICU. All recent commercial ICISs have incorporated CPOE, and some systems combine this with varying degrees of clinical decision support systems (CDSSs). Only a few authors have studied the impact of CPOE in the ICU, and even less have investigated the occurrence of medication prescription errors before and after the implementation of an ICIS [22,24-27]. A recent article by Shulman and colleagues [22] showed that CPOE without CDSS was able to eliminate many of the minor errors, but introduced new, potentially more serious errors in their ICU.

In one unit of our ICU, we implemented an ICIS with incorporated CPOE and a moderate level of CDSS. The objective of this study was to evaluate and compare the incidence and severity of medication prescribing errors (MPEs) between this CPOE unit and paper-based units.

## Materials and methods

### Setting

The study was conducted in a tertiary care University Hospital over a five week period (21 March to 28 April, 2004). The 22-bed surgical ICU was divided into three adjacent units of 8, 6 and 8 beds.

### Study design

A prospective, controlled cross-sectional trial was conducted in two paper-based units (PB-Us; total of 14 beds (8 + 6)) versus one computerized unit (C-U; 8 beds), 10 months after implementation of the ICIS in the latter unit. Patients were randomly assigned to either of these units by an independent nurse. All units had a similar case mix of patients. Medical staff, consisting of five senior intensivists and three residents, rotated continuously over these units, usually on a one-week basis. One month after the completion of the study, the ICIS was implemented in the two other remaining units. Approval of the ethics committee was obtained; informed consent was waived.

A surgical ICU-independent clinical pharmacist with experience in medication errors analyzed every medication order of randomly selected patients during this five week period and recorded every possible MPE. Physicians and nursing staff at the units were completely unaware of the ongoing study. As it was not possible to screen every patient on a daily basis because of lack of time, patients were picked with a minimal pause of one day between selections. All medication and fluid prescriptions were checked for errors in:

1. Drug (brand or generic) name (illegible, abbreviations, wrong name).

2. Dosing (overdose, underdose, dose omitted).

3. Dosage interval (incorrect dosage interval, dosage interval omitted).

4. Pharmaceutical form.

5. Preparation instructions (incorrect or omitted solvent or dilution, if not available on standard nursing charts).

6. Adequate drug monitoring (no monitoring, wrong drug monitoring, if necessary according to normal hospital practice).

7. Route of administration (incorrect route, route omitted).

8. Infusion rate of continuous medication (wrong rate, rate omitted).

9. Double prescriptions.

10. Clinically important drug-drug interactions.

11. Contra-indications to the prescribed drug.

12. Known allergy to the prescribed drug.

The appropriateness of drug choice was not considered. Transcription errors in the PB-U were taken into account. The pharmacist retrieved information out of the medical and nursing file and the laboratory data. Renal function was noted for every patient and renal failure was defined as calculated creatinine clearance less than 50 ml/minute. The parameters needed to calculate the creatinine clearance were always available in both the PB-U and the C-U. In addition to the pharmacists' own professional knowledge, clinical guidelines (Up to Date^®^, Waltham, MA, USA) and an interaction data bank (Thomson Micromedex^®^, Greenwood Village, USA, and Physician Desk Reference^® ^2003, USA) were used. Errors were identified within 24 hours after prescription, and further classified into different types, categories and possible causes, according to the National Coordinating Council for Medication Error Reporting and Prevention (NCC MERP) guidelines, which provide a standard language for reporting medication errors [28]. Classification of level of severity of medication errors occurred according to an adjusted numeric scaling system (based on the NCC MERP taxonomy) [28,29]. The NCC MERP severity classification was modified, since this classification is adequate for administration errors, but not entirely for prescription errors.

An independent panel, consisting of one clinical pharmacist, not involved in the registration part of the study, and two intensive care specialists, evaluated independently the severity of MPEs at least one month after screening. The panel was blinded for specific patient characteristics, as well as for patient group assignment. If agreement was not achieved during the first review, the three panel members discussed the incident until they reached consensus.

The description of groups according to level of severity of MPE is shown in Table 1. We identified three groups: minor MPEs (no potential to cause harm); intercepted MPEs (potential to cause harm but intercepted on time); and serious MPEs (non-intercepted potential adverse drug event (ADE) or ADEs, being MPEs with potential to cause, or actually causing patient harm).

### Description of the ICIS

The implemented system concerned an ICIS with incorporated CPOE and a moderate level of CDSS (Centricity Critical Care Clinisoft, GE Healthcare Europe, Helsinki, Finland), with full connections to monitors, ventilators, syringe pumps and also connection with the hospital information system for administrative patient data and laboratory results. The CDSS consisted of several different functionalities. There was a possibility for facilitated medication prescription by means of protocols for specific patient groups, for example, liver transplant patients or neurotrauma patients, with separate protocols for subgroups with renal failure or sedation. When choosing a drug, the most commonly used prescription with corresponding drug dose was shown, together with the different dosing schemes for renal insufficient patients (according to creatinine clearance, intermittent or continuous hemodialysis) and for patients with severe liver dysfunction. All these prescriptions had a fully preconfigured template. Clinically important interactions of commonly prescribed medication appeared at the time of prescription as pop-ups. Physicians were also notified about a number of important and possibly life-threatening drug-related complications (for example, QT interval changes with erythromycin). The allergy status of the patient was shown by means of a differentially colored highlighted icon in the toolbar as well as in the general prescription window. Sophisticated CDSS in the form of real-time alerts notifying the physician to adjust drug dosages to changing organ failure was lacking.

### Statistical analysis

The primary outcome measure was the difference in incidence and severity of MPEs in the C-U versus the PB-U. Secondary endpoints were univariate correlations between patient characteristics (APACHE II, renal failure, number of drug prescriptions (at screening day) and the number of MPEs.

Nonparametric data were analyzed with the Kruskal-Wallis and Mann-Whitney *U* tests. These data are presented as median values (with 25th and 75th percentiles). Nominal data were compared by using chi-square analysis or by Fisher's exact test as appropriate. Correlations between continuous variables were calculated by the Spearman rank correlation test. All reported tests are calculated two-tailed, and *P *< 0.05 was predetermined to represent statistical significance. All statistical analyses were carried out with SPSS 12.0 (SPSS Inc., Chicago, IL, USA).

## Results

During the five week study period we analyzed 160 patient-days in 90 different patients. Both the C-U and the PB-U group contained 80 patient-days. Patient characteristics are shown in Table 2.

A total of 2,510 medication and fluid prescriptions were evaluated by the clinical pharmacist, comprising 1,286 in the C-U and 1,224 in the PB-U. In the C-U, 44 MPEs occurred versus 331 in the PB-U (3.4% versus 27.0%, *P *< 0.001). Overall, the ICIS resulted in a relative reduction of 86.7% for all types of errors associated with medication ordering. These results are shown in Table 3.

In the C-U, the minor MPEs were mainly wrong pharmaceutical form errors and infusion rate errors. The intercepted MPEs particularly involved double prescriptions, but also problems with trailed zeros (for example, aspirin 3 g instead of 0.3 g), and problems with continuous infusion prescriptions (for example, propofol or remifentanil infusion being still activated two days post extubation). Another example of intercepted MPE involved the wrong prescription of a tenfold overdose of a beta-blocker, where rapid intervention of the clinical pharmacist intercepted the administration of this overdose. The non-intercepted potential ADEs were mainly dosing errors or incompleteness of low molecular weight heparin prescriptions. The two ADEs that occurred in the C-U involved an antibiotic overdose (level 2) and a vasopressin infusion overdose causing cardiac ischemia (level 3.5).

In the PB-U, there were many minor MPEs, mainly because of illegible writing, incomplete orders, or abbreviations. The intercepted MPEs were mostly errors of negligence (for example, wrong route of administration) or transcription errors. The ADEs were mainly dosing errors (especially for antibiotics and anti-epileptic drugs).

For patients with renal failure, a three-fold reduction of serious MPEs in the C-U versus the PB-U (12 versus 35, respectively; *P* < 0.001) was observed. In the PB-U, 91% of these serious MPEs were due to dosing errors, which is significantly higher than the proportion of dosing errors in the C-U (41%, *P* < 0.001).

**Figure 1:**
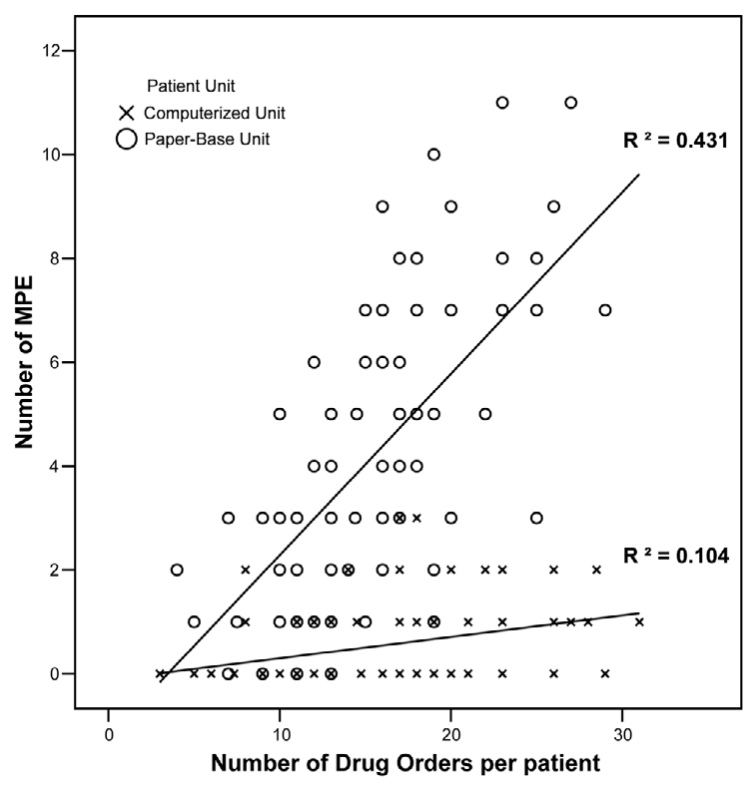
Scatter plot of number of medication prescribing errors (MPEs) at screening day according to number of drug orders per patient (24 hour screening day).

In the PB-U we observed a trend toward more prescription errors with increasing number of drug orders per patient (Figure 1). In contrast, in the C-U there did not seem to be a higher risk for errors if the amount of drug orders increased. This suggests that using the CPOE system can protect against MPEs in patients with multiple drug prescriptions.

**Figure 2:**
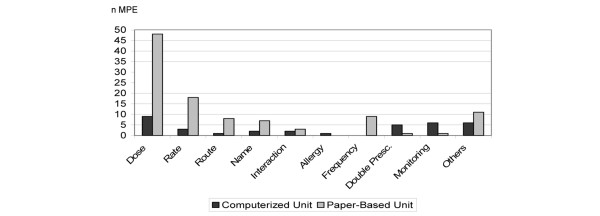
Types of intercepted medication prescribing errors (MPEs) and serious MPEs. Dose, dosing error; Rate, wrong infusion rate of continuous medication; Route, wrong route of administration; Name, error in drug name; Interaction, drug-drug interaction error; Allergy, known allergy to prescribed drug; Double presc., double prescriptions; Monitoring, drug monitoring error; Others, errors in posology, concentration, contra-indication.

Types of intercepted and serious MPEs (level 0.5 to 6) are shown in Figure 2. The dosing errors were the most frequent type of errors in both groups, followed by double prescription and drug monitoring errors in the C-U. These last two errors were rarely seen in the PB-U, which means double prescriptions and drug monitoring errors were new errors resulting from the computerized system. All double prescription errors, in both groups, were minor or intercepted MPEs, whereas the drug monitoring errors were also classified as non-intercepted potential ADEs (C-U, five out of eight; PB-U, one out of two).

The most common drug classes associated with intercepted and serious MPEs were antibiotics (PB-U, 23.5% (*n* = 25); C-U, 23% (*n* = 8)), cardiovascular medication (PB-U, 23% (*n* = 24); C-U, 37% (*n* = 13)) and sedatives (PB-U, 19.8% (*n* = 21); C-U 12.5% (*n* = 4)).

## Discussion

To our knowledge, this is the first study evaluating the effect of CPOE (with a moderate level of CDSS) on MPE's simultaneously in a paper-based and an already computerized ICU. Most other articles studying the impact of CPOE on MPEs have a before-after design, which induces an important bias in time [7,15,17,22,30,31]. Additionally, some of these studies investigated the implementation of a CPOE system, not a full computerized ICU system with connection to all monitors, ventilators and the hospital information system [7,15].

Our study, like others, shows that CPOE has the potential to almost completely eliminate minor MPEs [17,32]. The incidence of minor MPEs decreased from 18.3% in the PB-U to 0.7% in the C-U, since completeness and legibility of the order was mandatory in the CPOE part. However, a missing infusion rate was still allowed by the system, which caused a few minor MPEs in the C-U. The wrong pharmaceutical form errors were configuration errors, which have been adjusted after the study. Because these minor MPEs are not harmful, and do not place a great burden on patient safety, they are not discussed in detail.

The incidence of intercepted MPEs was four times lower in the C-U than in the PB-U. A few of these errors concerned problems with trailed zeros, but most of them were double prescriptions, which were identified by the nurse or the physician. These types of errors did not occur in the PB-U, meaning they were caused by the CPOE system itself. But as these errors did not reach the patient, we choose not to assign a severity level. This is in contrast to the study of Shulman and colleagues [22], who rated not only non-intercepted but also the intercepted errors. Two out of the three major intercepted errors they described could not have happened with our system. For every medication, very detailed predefined standardized drug dosage regimens were created in our CPOE, thereby limiting the need to adjust a chosen drug prescription and eliminating the use of pull down menus. For example, in the case of vancomycin prescriptions, physicians had to order a 'vancomycin loading dose' and a 'vancomycin dose according to plasma level', without having to adjust anything, which virtually eliminates the risk of making errors.

Regarding the intercepted and serious MPEs, we observed a 67% decrease, which is similar to several other studies that reported decreases of 55% to 86% [7,15]. Many patients in the PB-U experienced at least one intercepted or serious MPE in comparison to patients in the C-U (67.5% versus 32.5%, respectively).

The amount of ADEs was significantly reduced by the CPOE. The two ADEs that did occur in the C-U could not have been avoided by our current CPOE and moderate level of CDSS. Comparison between studies remains difficult because there is no consensus for medication error classification. But when we compare our results with those of Shulman and colleagues [22], we do find some important differences. Firstly, we found a significant reduction in dosing errors in the C-U, whereas Shulman and colleagues found a higher proportion of dosing errors in the CPOE group. This could be partially explained by our method of drug ordering, which virtually eliminates the need for adjustments in the prescription window. Besides being a comfortable way of prescribing, it is also less time-consuming. Secondly, CPOE caused many minor errors with no harm, similar to what we found, but they also found many errors requiring more monitoring. In our study, we only found two of those errors (classified as ADE level 2) as the ICU is already a highly monitored environment. Thirdly, in Shulman and colleagues' study, prescriptions that were not signed were regarded as a medication error (33.3% of the CPOE errors). This was not the case in our study, as the ICIS demands a password for prescribing a drug, meaning that every prescription is electronically signed.

We believe that our estimate of reducing medication errors in the ICU by implementing a CPOE is conservative. First, there could be a bias since the physicians working in the C-U as well as in the PB-U had an opportunity to learn how to prescribe a drug correctly (adjusted to renal or hepatic function), which can account for a lower incidence and severity of MPEs in the PB-U. Secondly, this study only investigated prescription errors, and not dispensing or administration errors. Administration errors are the second most frequent cause of medication errors, but are rarely studied in the ICU [33-35]. ICIS provides many advanced features to prevent errors in the administration process by showing important information to the nursing staff regarding administration procedures and safety.

As in our study, it already has been shown previously that CPOE can create new problems, such as inconsistent or duplicate orders [22,36,37]. Causes were related to deficiencies in the CPOE system itself or to human shortcoming (for example, physicians bypassing the normal way of prescribing). By performing this study, however, we identified problems within the CPOE system and were able to correct them. The following examples show that it is very important to objectively evaluate a newly installed system and correct the problems you encounter. The first example of a frequent error was the unnoticed changing of an already activated prescription of a continuous infusion medication. Since recent upgrading of the system every continuous infusion prescription change becomes immediately visible by adding a black sign. Another problem was the request of drug plasma concentration levels. They were often being forgotten or, on the other hand, still asked for when the medication had already been stopped. The problem lies in the rigidity of the system to electronically prescribe the laboratory item: physicians had to request the laboratory orders on a daily basis and, in contrast to the paper chart, it was not easy to see which laboratory orders were made the previous day. Once the study was finished, we configured a more elegant way of laboratory requesting by means of protocolized laboratory order requests.

The allergy notation was properly filled in 69% of the patients in the C-U, whereas only 2% had an allergy notation in the charts of the PB-U. The only allergy error we encountered was in the C-U in a patient whose allergy status was not noted in the ICIS, although it was clearly notified in the patient charts. The study, however, was conducted four weeks after an upgrade with installation of the allergy notification, and a recent evaluation showed a more adequate registration. Our study, however, has several limitations. First, the study took place at only one tertiary care teaching hospital. The effect of CPOE on the incidence of MPEs depends on the implemented system; therefore, our results may not be generalized to other ICU settings and other ICISs.

Secondly, the absolute numbers of ADEs in both our groups (C-U and PB-U) are higher than those reported in previous trials [11,38]. In the C-U, the incidence of ADEs was 25 events per 1,000 patient-days, whereas in the PB-U it was 150 events per 1,000 patient-days. In other studies, however, the amount of ADEs was 10.4 [38] to 19 events per 1,000 patient-days [11]. The fact that this study was conducted in a teaching ICU could explain this higher number [39]. A second explanation could be the number and complexity of medication prescriptions, which increase the occurrence of MPEs. This has also been previously shown by Cullen and colleagues [11], who saw a higher rate of preventable potential ADEs in ICU settings. But when adjusting for the number of drugs ordered, he found no differences in error rates between ICU and non-ICU. A third explanation for this higher rate of ADEs could be the detection method for medication errors. Most studies involving medication errors and ADEs in the ICU are retrospective chart reviews (mostly by trained nurses) and/or self-report studies [3,11,40]. This latter technique is likely to underestimate the true incidence of medication prescribing errors [41,42]. In our study, chart review was done prospectively by the clinical pharmacist, who typically found higher rates of ADEs [43-45]. Additionally, the case finding could be facilitated by the CPOE system itself, as has been recently shown by Nebeker and colleagues [42], who also found higher rates of medication errors than those reported in the literature. Finally, it is possible that the paper chart, which was prepared by a resident in advance, contained more mistakes because the medication file was not adjusted to the clinical status of the patient overnight, and because of negligence or high work pressure.

Another potential bias in this study could be that some patients were at least double screened (17 patients in the C-U, 18 patients in the PB-U). However, no patient was screened on two consecutive days. In the C-U, one identical non-intercepted potential ADE occurred in a patient who was screened with an interval of three days. In the PB-U, four patients had at least one completely identical medication error, with a total of eight identical MPEs. Of these errors, there was one intercepted MPE, three non-intercepted potential ADEs, and four minor MPEs. Finally, although rotating physicians and nurses were unaware of the study registration by the clinical pharmacist, we cannot exclude the possibility that some bias may have resulted from some interventions that were made by the clinical pharmacist to prevent a potentially serious or life threatening error to occur.

## Conclusion

Implementation of CPOE with a moderate level of CDSS showed a significant reduction in incidence and severity of MPEs, and significance was found through all levels of severity. However, CPOE had the highest potential to eliminate MPEs at the lowest level of severity. Furthermore, evaluation of the CPOE enabled us to identify newly introduced problems, and gave us the opportunity to take corrective actions.

This study once again underscores the importance of evaluating newly installed systems, even if it is a vendor-built product. To be able to compare different studies, it would be of great benefit to have a more standardized way of error classification and detection. This would substantially simplify the discussion about whether CPOE alone, or with a varying degree of CDSS, is a more or less effective way of improving quality of care.

## Key messages

• This is the first ICU study with a prospective cross-sectional design evaluating MPEs and ADEs in a CPOE unit versus a paper-based unit.

• CPOE had the highest potential to eliminate MPEs at the lowest level of severity but also the amount of ADEs was significantly reduced.

• For patients with renal failure, a three-fold reduction of serious MPEs with CPOE was observed, mainly by reducing dosing errors

• By evaluation of the ICIS we identified newly introduced problems, and were able to take corrective actions.

## Abbreviations

ADE = adverse drug event; CDSS = clinician decision support system; CPOE = computerized physician order entry; C-U = computerized unit; ICIS = intensive care information system; ICU = intensive care unit; MPE = medication prescribing error; NCC MERP = National Coordinating Council for Medication Error Reporting and Prevention; PB-U = paper-based unit.

## Competing interests

The authors declare that they have no competing interests.

## Authors' contributions

All of the authors were involved in designing the study. KC was responsible for conceiving the study, data acquisition, analysis of the data, statistical analysis and drafting of the manuscript. BC was responsible for data acquisition, analysis of the data, and drafting of the manuscript. JD was responsible for conceiving the study, statistical analysis and critical revision of the manuscript. AS, KV and HR were responsible for critical revision of the manuscript. All authors read and approved the final manuscript.

## Figures and Tables

**Table 1:** Descriptions of level of severity of medication prescription errors

Major divisions	Numeric scale	Description (NCC MERP scale)
Minor MPE	0	Incomplete order, no potential to cause harm (A)
Intercepted MPE	0,5	Potential error, intercepted, error did not reach the patient (B)
Serious MPE		
N-I potential ADE	1	Error reached the patient, but caused no harm (C)
ADE	2	Error occurred, resulted in increased patient monitoring, but no harm to the patient (D)
	3	Error occurred with change in vital signs, increased need of monitoring or laboratory tests, eventually no harm (D)
	3.5	Error occurred with temporary harm, needing treatment/intervention (E)
	4	Error occurred with temporary harm, needing treatment with another drug, increased length of stay or required intervention to prevent permanent impairment or damage (F)
	5	Error occurred and resulted in permanent patient harm (G)
	5.5	Error occurred and resulted in near death event (H)
	6	Error occurred and resulted in patient death (I)

MPE, medication prescribing error (an error in the prescribing or monitoring of a drug); for example, an order written for aminoglycosides, without ordering a drug plasma level, or without a route of administration. Minor MPE: minor medication prescription error (an MPE that has no potential to cause harm); for example, an abbreviation or a missing route of administration. Intercepted MPE: an MPE that has the potential to cause patient harm but did not because the error was intercepted in time. N-I Potential ADE: non-intercepted potential ADE. ADE: adverse drug event; these are further specified according to level of severity (level 2 to 6). The N-I potential ADEs and ADEs consist of serious errors because they have the potential to or actually cause injury and, therefore, are the most important from the perspective of patient safety. For this reason, these two groups are joined into one serious MPE group. The National Coordinating Council for Medication Error Reporting and Prevention (NCC MERP) scale is mentioned for comparative purposes.

**Table 2:** Patient characteristics

Characteristic	C-U (80 patient-days)	PB-U (80 patient-days)	*P*
Age (years)	61.5 (45–71)	54 (37–65)	0.021
Drug prescriptions	17 (11–20)	15 (12.25–18)	0.386
APACHE II	20 (15–30)	20 (16–24)	0.275
SOFA	5 (3–9)	6 (4–8)	0.267
Renal failure (%)	31.2	37.5	0.407
LOS	2 (1–8)	5 (2–9)	0.016

Data are expressed as median with interquartile range in parentheses. Drug prescriptions is the number of drug prescriptions at screening day. APACHE II is the acute physiology and chronic health evaluation score at day 0. SOFA is the sepsis-related organ failure assessment score at screening day. Renal failure is creatinine clearance <50 ml/minute. LOS, length of stay at screening day. C-U, computerized unit; PB-U, paper-based unit.

**Table 3:** Medication prescription error analysis in computerized and paper-based units

	Computerized unit	Paper-based unit	*P*
Total prescriptions (*n*)	1,286	1,224	NS
Total MPEs (*n*)	44	331	<0.001
% MPEs	3.4	27.0	<0.001
Minor MPEs	9	225	<0.001
Per 100 orders	0.7	18	
Intercepted MPEs (*n*)	12	46	<0.001
Per 100 orders	0.9	3.8	
Non-intercepted potential ADEs (n)	21	48	<0.001
Per 100 orders	1.6	3.9	
Total ADEs (*n*)	2	12	<0.01
Per 100 orders	0.15	1.0	
Intercepted MPEs and serious MPEs	35	106	<0.001
Serious MPEs	23	60	<0.001

ADE, adverse drug event; MPE, medication prescription error; NS, not significant.
